# Biosynthesis of Gold Nanoparticles Using *Fusarium oxysporum* f. sp. *cubense* JT1, a Plant Pathogenic Fungus

**DOI:** 10.5402/2013/515091

**Published:** 2012-11-11

**Authors:** Janki N. Thakker, Pranay Dalwadi, Pinakin C. Dhandhukia

**Affiliations:** ^1^Department of Biotechnology, P. D. Patel Institute of Applied Science, Charotar University of Science & Technology, Education Campus Changa, Changa 388421, India; ^2^Ashok and Rita Patel Institute of Integrated Study & Research in Biotechnology and Allied Sciences, New Vallabh Vidyanagar 388 121, India

## Abstract

The development of reliable processes for the synthesis of gold nanoparticles is an important aspect of current nanotechnology research. Recently, reports are published on the extracellular as well as intracellular biosynthesis of gold nanoparticles using microorganisms. However, these methods of synthesis are rather slow. In present study, rapid and extracellular synthesis of gold nanoparticles using a plant pathogenic fungus *F. oxysporum* f. sp. *cubense* JT1 (FocJT1) is reported. Incubation of FocJT1 mycelium with auric chloride solution produces gold nanoparticles in 60 min. Gold nanoparticles were characterized by UV-Vis spectroscopy, FTIR, and particle size analysis. The particles synthesized were of 22 nm sized, capped by proteins, and posed antimicrobial activity against *Pseudomonas* sp.

## 1. Introduction

Metal nanoparticles exhibit unique electronic, magnetic, catalytic, and optical properties that are different from bulk metals and dependent on their size and shape [1–5]. Gold is often considered the most inert of all metals; however, methods for preparing catalysts having nanoparticles of gold on oxide supports have opened up this new area of opportunity [6]. Gold nanoparticles are of interest mainly due to their stability under atmospheric conditions, resistance to oxidation, and biocompatibility [7, 8]. Therefore, development of techniques for synthesis of gold nanoparticles, of well-defined size and shape, is of great challenge. Different chemical methods developed to control the physical properties of the particles for their different applications. Most of these methods are still in the development stage, and problems are often experienced with stability of the nanoparticle preparations, control of the crystal growth, and aggregation of the particles [9, 10]. There is an increasing pressure to develop clean, nontoxic, and environmentally benign synthetic technologies. Microbial resistance against heavy metal ions has been exploited for biological metal recovery via reduction of the metal ions or formation of metal sulfides [7]. Recently, microorganisms such as bacteria and fungi were shown to be attractive alternative to synthesize gold nanoparticles [11, 12]. However, there is a limited amount of information on the extracellular biosynthesis of gold nanoparticles. 

Metal nanoparticle synthesis depends on the reducing agent, which reduces Au^3+^ in to Au^0^ state [12]. During plant-pathogen interaction, plants are known to produce reactive oxygen species as a defense mechanism against pathogens. For successful infection, pathogen must have high reducing capabilities to counteract plant's defense. Therefore, plant pathogenic organism could be an excellent source for generation of extracellular metal nanoparticles. A plant pathogenic fungus, *Fusarium oxysporum *f. sp. *cubense* JT1 (FocJT1) isolated from infected banana plants causing Panama disease was characterized for its ability to cause successful infection in previous studies [13, 14]. Therefore, present study was aimed to access the ability of a plant pathogenic fungus FocJT1 and to reduce Au^3+^ ions extracellular at room temperature with a single-step process for synthesis of gold nanoparticles. 

## 2. Materials and Methods

### 2.1. Microorganism

Previously isolated *Fusarium oxysporum *f. sp. *cubense *JT1 (FocJT1) was maintained on potato dextrose agar (PDA) at 4°C. Mycelial fragments were transferred from PDA slant to PDA plates and incubated for four days at 27°C. 

### 2.2. Cultural Technique

Using a sterile cork borer, 8 mm agar plug was cut from four-day old culture and inoculated in 250 mL Erlenmeyer flasks containing 100 mL of PDB. The flasks were incubated at 27°C for 21 days in static condition. For analyzing biosynthesis of gold nanoparticles by FocJT1, one set was augmented with 10 mM final concentration of HAuCl_4_ and other served as control. Periodically, samples were withdrawn and analyzed for nanoparticle synthesis.

### 2.3. Microscopic Observation of Biomass from Control and Experimental Flasks

Mycelial fragments from control and experimental flasks were kept on a clean glass slide and covered with cover slip. Microscopic observations for difference in both were recorded using Olympus CH20i Microscope (BIMF, CIAS).

### 2.4. UV-Visible Spectroscopy

Samples were collected at time intervals of 0 min, 30 min, 60 min, 90 min, 24 h, and 48 h from experimental and control flasks. Synthesis of gold nanoparticles was analyzed using UV-visible spectroscopy (Cyberlab spectrophotometer) operated at a resolution of 1 nm from 340 to 700 nm range in a 1 cm path quartz cell.

### 2.5. Fourier Transform Infrared Spectrometry

KBr Pellets with mycelial mat of control and experimental were prepared. Pellets formed were analyzed with FTIR (NICOLET Thermo Scientific) spectroscopy at K. C. Patel R&D Center, CHARUSAT, Changa, India.

### 2.6. Particle Size Analysis

For particle size analysis of gold nanoparticles, the gold nanoparticle-fungus reaction solution was filtered with Whatman filter paper followed by filtration using 0.2 *μ* filter to remove any spores or mycelia. Filtrate collected was dried, scraped with scalpel, and suspended in ethanol. Particle size was determined using Particle Size Analyzer (MALVERN Instruments) at K. C. Patel R&D center, CHARUSAT, Changa, India.

### 2.7. Antibacterial Activity

Antibacterial activity of gold nanoparticles was evaluated against Gram negative test organism *Pseudomonas *sp. In sterile condition, prior sterilized and dried cotton cloth (2 × 2 cm) was immersed in flask containing FocJT1and FocJT1 augmented with HAuCl_4_ for one hour at room temperature. The cloth was collected and dried. From overnight grown culture of *Pseudomonas *sp., 0.1 mL was spread on PDA plates and allowed to dry for 10 min. Au nanoparticle impregnated cloth was kept in the centre of inoculated plate and incubated at 37°C for 24 hours. Plates were observed for zone of inhibition.

## 3. Results and Discussion

### 3.1. Gold Reduction

Plant pathogenic fungus, FocJT1, isolated from wilt infected banana plants, was investigated for its ability to synthesize gold nanoparticles. Change in appearance of mycelia and culture filtrate was observed by incubating mycelial mat with auric chloride solution.

**Figure 1:**
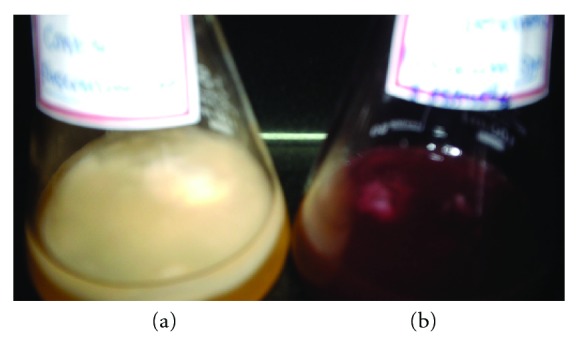
(a) FocJT1 control and (b) incubated with the 10 mM HAuCl_4_ solution.

The mycelium exhibited a pale yellow color before reaction with the auric ion, which changed to a purple color upon completion of the reaction (Figure 1). Appearance of a burgundy red color in solution containing the biomass was a clear indication of the formation of gold nanoparticles in the reaction mixture and was due to the excitation of surface plasmon vibrations in the nanoparticles [15].

Reports on several hydroquinones with excellent redox properties that could act as electron shuttles in metal reductions are in [16, 17]. Thus, evidently electron shuttles or other reducing agents released by Foc are capable of reducing gold ions to gold nanoparticles.

Additionally, metal reduction ability is strain-specific. Ahmad et al. [15] suggested that reductase specific to *F. oxysporum *and prolonged reaction of Ag^+^ ions with another fungus, *F. moniliforme*, did not result in the formation of silver nanoparticles, neither intracellularly nor extracellular.

### 3.2. UV-Visible Spectroscopy

Gold nanoparticles exhibit new optical properties, which are not observed neither in molecules nor in the bulk metals [18]. One example is the presence of absorption band in visible region. This band appears due to the surface plasmon-oscillation modes of conduction electrons, which coupled through the surface to external electromagnetic fields [19]. The surface plasmon resonance and large effective scattering cross-section of individual metal nanoparticles make them ideal candidate for molecular labeling [20].

**Figure 2:**
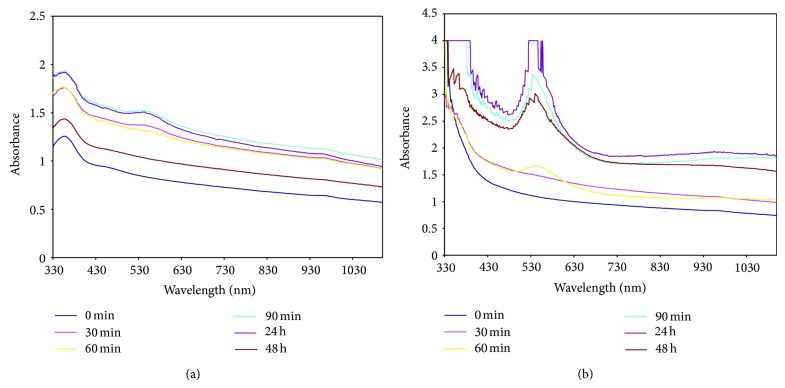
UV-visible spectra recorded as a function of time of reaction of control with fungal biomass in (a) media and (b) media augmented with 10 mM HAuCl_4_.

Therefore, synthesis of gold nanoparticles was assayed using spectral scan by UV-visible spectroscopy in the spectral region 340–700 nm. This technique outlined above has proved to be very useful for the analysis of nanoparticles [21]. As illustrated in Figure 2(a), the UV-Visible spectra recorded as a function of time of reaction of control having fungal biomass in medium (control) whereas Figure 2(b) shows the UV-Vis spectra of an aqueous solution of 10 mM HAuCl_4_ with the fungal biomass and medium. A broad peak located between 500 and 580 nm was found to increase with time representing the gold nanoparticles synthesis. The peak was developed in 60 min of reaction sample indicating that the synthesis of gold nanoparticles starts rapidly using FocJT1. The peak reached to near saturation at 90 min reaction time. In earlier studies on the synthesis of silver and gold nanoparticles using bacteria [11] and fungi [12], the time required for completion of the reaction (i.e., complete reduction of the metal ions) ranges from 24 to 120 h.

### 3.3. FTIR Spectra of Gold Nanoparticles

**Figure 3:**
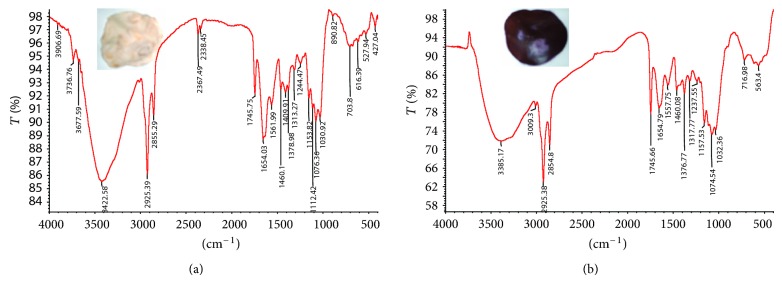
FTIR spectra of (a) dried mycelial mat and (b) mycelia mat incubated with 10 mM HAuCl_4_ solution. Mycelia mat collected are shown in inset.

FTIR measurement carried out shows the amide bands, which were due to –N–H stretch and carbonyl stretch vibration in the amide linkages of the protein. The bands at 1634 and 1560 cm^−1^ were identified as the amide I and II bands [22] and arise due to carbonyl stretch and –N–H stretch vibrations in the amide linkages of the proteins, respectively [23]. The positions of these bands are close to those reported for native proteins. The FTIR results thus indicate that secondary structure of the proteins is not affected because of reaction with the Au^3+^ ions or binding with the gold nanoparticles. The band at ca. 1458 cm^−1^ is assigned to methylene scissoring vibrations from the proteins in the solution (Figure 3). On comparison of the IR spectra of FocJT1 control and experimental mat with gold nanoparticle, it was observed that 1654 and 1460 cm^−1^ bands were masked. This indicated that the gold particle formed were in conjugation with protein. Intensity of band 3385 cm^−1^ was reduced indicating that –OH stretching of alcohol and phenol present on the surface of the mycelial mat might be involved either in the formation or the attachment.

### 3.4. Particle Size Analysis

**Figure 4:**
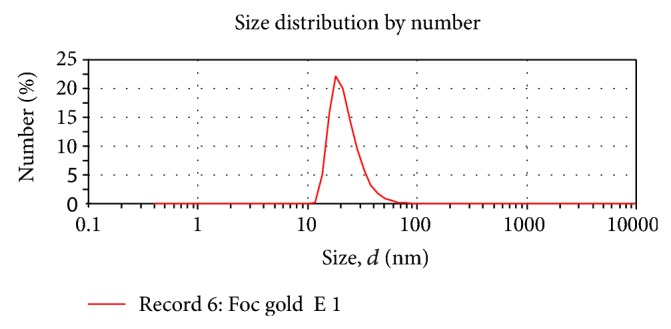
Particle size distribution of the biosynthesized gold nanoparticles.

Biosynthesized gold nanoparticles were found to be of 22 nm size using particle size analyzer (Figure 4). Extracellular reduction of the metal ions by Foc resulted in the rapid formation of the highly stable gold nanoparticles of 22 nm dimensions. The nanoparticles were not in direct contact within the aggregates, indicating stabilization of the nanoparticles by capping agents. The solution of gold nanoparticles synthesized by the reaction of Au^3+^ ions with FocJT1 was exceptionally stable.

### 3.5. Antimicrobial Assay

**Figure 5:**
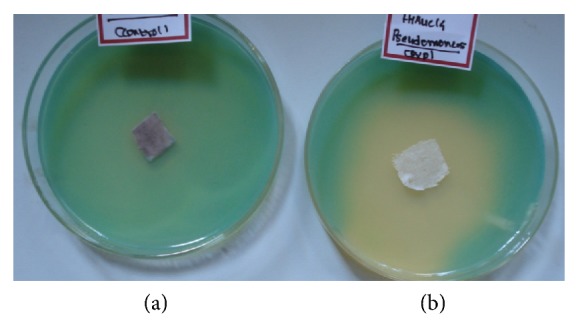
Antimicrobial activity of gold nanoparticles coated on cotton cloth against *Pseudomonas* sp. (a) control (b) experimental.

Gold nanoparticles synthesized by FocJT1 strain incorporated in cotton cloth exhibited antibacterial activity against *Pseudomonas* sp. (Figure 5). The mechanism of the inhibitory effects of Au ions on microorganisms is partially known. Some studies have reported that the positive charge on the Au ion is crucial for its antimicrobial activity through the electrostatic attraction between negative-charged cell membrane of microorganism and positive-charged nanoparticles [24]. 

## 4. Conclusion

The present study proved a rapid and extracellular biosynthesis of gold nanoparticles by a fungus, FocJT1, isolated from wilt infected banana plants. Upon addition of the gold ion (10 mM) into the flask containing the mycelial mat, the color of the medium changed very rapidly to burgundy red, which could be due to the excitation of surface plasmon vibrations, typical of the gold nanoparticles. This indicated the presence of gold nanoparticles. The FTIR spectra suggested the capping of gold nanoparticles by protein. A biological process with the ability to strictly control the shape of the particles produced would therefore be an exciting prospect. However, the cellular mechanism leading to the biosynthesis of gold nanoparticles is not yet fully understood. Further research will therefore focus on the development of a fundamental understanding of the process mechanism on -cellular and molecular levels, including isolation and identification of the compounds responsible for the reduction of auric ions.
